# Effect of asthma education intervention on self-management knowledge and control level in Tigray, Northern Ethiopia: a quasi experimental study

**DOI:** 10.1186/s12890-025-03574-4

**Published:** 2025-03-15

**Authors:** Tirhas Gebremedhin Gebresilassie, Alemayehu Worku, Ahmed Ali Ahmed, Negussie Deyessa Kabeta

**Affiliations:** 1 Department of Public Health, College of Health Sciences, Aksum University, P.O.Box: 298, Axum, Tigray Ethiopia; 2 School of Public Health, College of Medicine and Health Sciences, Addis Ababa University, Addis Ababa, Ethiopia

**Keywords:** Asthma, Control level, Education, Self-management knowledge, Intervention

## Abstract

**Background:**

Asthma self-management education empowers patients to manage their condition effectively. However, evidence on its impact in Ethiopia remains limited. This study evaluated the effect of asthma education on asthma control and self-management knowledge among adult asthma patients in Ethiopia.

**Methods:**

A quasi-experimental design was employed, with a total of 204 participants, comprising 102 individuals in the intervention group and 102 in the control group at baseline. After accounting for follow-up losses (20.6% in the intervention group and 23.5% in the control group), 81 participants from the intervention group and 78 from the control group were retained six months after the completion of the education (post-intervention). Pre- and post-intervention assessments were conducted using validated questionnaires to measure asthma control levels and self-management knowledge. The intervention group received a structured, small-group asthma education program comprising three sessions over six months. The intervention's effect was analyzed using linear regression models for difference-in-differences and interaction effects, while heterogeneity analysis was performed using a generalized linear model.

**Results:**

10% of the intervention group and 7.8% of the control group reported prior asthma management education, most of which (60%) was over a decade ago. Asthma control levels significantly improved in the intervention group, with a 19.4% increase compared to 0.6% in the control group. The overall increase in the intervention group was 18.8% higher than in the control group (*P* = 0.03). Similarly, self-management knowledge improved markedly in the intervention group, with a 24.3% increase compared to 0.7% in the control group. The intervention group demonstrated a 23.6% overall improvement relative to the control group (*P* = 0.000).Participants in the intervention group were six times more likely to achieve well-controlled asthma and 13 times more likely to exhibit good self-management knowledge compared to the comparison group (*p* < 0.01). The intervention’s impact was consistent across subgroups, with no significant variations by socio-demographic and asthma related factors.

**Conclusions:**

Asthma self-management education interventions significantly enhance asthma management knowledge and control levels. This study highlights the need to implement and expand asthma education programs during patient follow-ups to empower patients, to reduce medical costs, unscheduled hospital visits, emergency department visits, and premature mortality.

**Trial registration:**

Registered retrospectively with TRN PACTR202407741896902.

**Supplementary Information:**

The online version contains supplementary material available at 10.1186/s12890-025-03574-4.

## Background

Asthma is a chronic inflammatory lung disease affecting approximately 262 million people globally. It is associated with significant economic, social, and health-related burdens, including premature death and diminished quality of life (QOL). Asthma affects an estimated 8% of adults worldwide [1, 2]. In Ethiopia, current asthma prevalence studies report variable rates ranging from 4.9% to 29.6% [3–5].

The term self-management refers to a patient’s ability to adopt behaviors and routines that optimize their control over their illness. According to Knibb, Alviani, et al. [6], self-management empowers individuals with chronic conditions to acquire the knowledge and skills needed to make informed decisions about their care [6]. Patients who receive asthma self-management education (ASME) get the feeling of more in control of their health, are enabled to take charge of their treatment, and better understand the risks they may face. Due to frequent exacerbations and the long-term nature of the disease, asthma significantly impacts a patient’s QOL across numerous aspects [7].

Promoting asthma education for patients is essential for effective self-management and the prevention of exacerbations. Since patients are primarily responsible for managing their medication at home, they need knowledge of symptom recognition and the appropriate use of reliever medications. While healthcare providers prescribe reliever and controller medications, consistent use of controller medications and adherence to a comprehensive self-management plan mainly depend on the patient [8].

Providing patients comprehensive knowledge about asthma fosters a proactive approach to care. This not only improves overall health and QOL but also reduces the emotional, physical, and financial burdens of asthma on individuals and society. Empowered patients experience fewer complications, better treatment adherence, and significantly reduced reliance on emergency care and hospitalizations [7].

A number of studies suggest that asthma self-management education improves both asthma control levels [9, 10]and self-management knowledge [11–13]. Moreover the Global Initiative for Asthma (GINA) guidelines emphasize personalized asthma management, highlighting the regular use of inhaled corticosteroids, even for mild asthma, and reducing reliance on short-acting beta-agonists (SABAs). The guideline also emphasis on reducing exacerbation risks through early anti-inflammatory treatment, trigger avoidance and management, patient education, and action plans for controlling symptoms and preventing exacerbations [14–17].

The denial of a chronic illness, ignorance of its progression, inability to adhere to medication regimens, and lack of self-management skills are key factors contributing to severe asthma morbidity [17, 18]. Asthma has a substantial impact on patients' health, often leading to frequent flare-ups that worsen their overall well-being. The condition also imposes a heavy burden on families, the economy, and society, due to the need for frequent ER visits, hospital stays, increased medical expenses, and asthma-related mortality [14, 19, 20].

Emergency room (ER) visits and hospitalizations due to asthma remain a global challenge, underscoring the need for improved management strategies. In 2020, nearly 1 million people visited an emergency department (ED) for asthma-related care, and 94,560 individuals were hospitalized due to asthma [21]. Across sub-Saharan Africa, up to 25% of asthma patients require emergency care annually. Hospitalization rates often exceed 10 per 1,000 individuals in urban areas, exacerbated by air pollution and limited healthcare infrastructure [22, 23].

In Ethiopia, hospitalizations and ER visits are further compounded by limited healthcare access, under diagnosis, and environmental exposures. Asthma prevalence in Ethiopia is rising, driven by urbanization and environmental changes. A study conducted at Jimma University Specialized Hospital reported high rates of emergency visits among asthma patients, with a significant proportion linked to uncontrolled asthma and poor access to inhaled corticosteroids [24]. However, specific annual figures of hospitalization and emergency visit data remain limited.

The economic burden of asthma is immense, encompassing billions in healthcare costs, lost productivity, and reduced QOL. In the United States, asthma imposed an annual economic burden of $81.9 billion between 2008 and 2013, including healthcare expenses, missed work and school days, and mortality [25]. In Africa, asthma contributes to high out-of-pocket expenses for individuals and strains national economies. For instance, in Nigeria, the average annual cost of asthma care per child is $162.49, contributing to a national expenditure of $0.16 billion [26]. Across sub-Saharan Africa, the average annual direct cost per patient is approximately $368.40, with 87% of these costs borne directly by patients. Affordability of medications remains a critical issue, as inhalers can cost up to 20% of the average monthly income in some African countries [20, 23, 27].

Many studies worldwide have demonstrated that asthma self-management skills and practices can be significantly improved through patient education [28]. Consequently, patient education has become a cornerstone of asthma management. The growing prevalence of asthma, improved understanding of self-management, and increasing interest in educational theories have underlined the importance of educational interventions in asthma [29].

Among various educational approaches, repeated small-group asthma education is particularly effective. Small-group settings provide a collaborative environment where patients can discuss, share experiences, and reinforce their understanding of asthma management strategies. Evidence indicates that repeated exposure to such education improves asthma knowledge and self-management practices, encouraging sustained commitment to better asthma control [30, 31].

Evidence indicates that asthma self-management education improves knowledge among adult asthma patients, resulting in better disease control. In contrast, studies in low- and middle-income countries (LMICs) showed that limited asthma knowledge and misconceptions are key patient-related factors that negatively impact asthma control [20]. Another study on the impact of improper inhaler technique on asthma control found that a lack of education on and insufficient follow-up care were key factors leading to incorrect inhaler use. This shows the importance of providing patients with comprehensive self-management education to improve their inhaler technique and enhance asthma control. In Ethiopia currently, there is no documented evidence on insufficient education to improper inhaler technique or poor asthma control though other factors such as low income, presence of comorbid condition, asthma severity and use of SABA alone as anti-asthmatic medication contributing to uncontrolled asthma [24].

Several studies assessing asthma control levels in Ethiopia have shown that most asthma patients struggle with poor disease control. Specifically, studies from different regions have reported well-controlled asthma rates of 33.3% in the Tigray region, 24.2% in Addis Ababa, and 28.6% in Jimma town were found to have well-controlled asthma [32–34] which shows a significant challenge in asthma management, with a large proportion of patients struggling to achieve adequate disease control.

The existing literature reveals a significant gap in understanding how insufficient self-management education contributes to poor asthma control, with limited research on the effectiveness of such education in Ethiopia. This study aims to fill this gap by assessing the impact of asthma self-management sessions on asthma control and knowledge among adults in Ethiopia.

## Materials and methods

The study used a quasi-experimental design to assess the impact of asthma self-management education on patients' knowledge and asthma control, comparing outcomes between an intervention group (receiving the education) and a control group (not receiving the education).

### Study setting and period

The study involved 204 adult asthma patients from 12 hospitals in the Tigray region. It focused on individuals diagnosed with asthma, who were ambulatory and not hospitalized, and took place in various outpatient settings, including outpatient departments, chest clinics, and chronic care units. The study was conducted between May 2018 and September 2020.

### Sampling technique and selection criteria

To ensure an adequate sample size, purposive sampling was used to assign hospitals to the intervention and control groups, based on the variation in their catchment areas and the populations they serve. A two-phase sampling process was employed.

#### Phase 1

Two referral hospitals and 14 general hospitals were selected. Of these, two referral hospitals and 10 general hospitals were purposively assigned to either the intervention or control group. This purposive hospital assignment was based on the catchment areas they serve and the availability of asthma care services they provide.

#### Phase 2

After assigning hospitals to the intervention and control groups, Patients were conveniently sampled from assigned intervention and control hospitals to meet the required sample size. Between 13 and 16 patients from each hospital were recruited based on their arrival, with recruitment ceasing once the target was reached. Only those who continued until the post-test were included in the analysis. Participants were selected according to the following criteria:

#### Inclusion criteria

Adults aged 18 years and above, with a physician-confirmed diagnosis of asthma, attending a follow-up visit at chest clinics or outpatient departments in the selected hospitals, and willing to participate in the study and provide informed consent.

#### Exclusion criteria

Critically ill asthmatic patients requiring urgent medical care were excluded and referred for immediate treatment. Additionally, patients unable to participate due to severe illness or cognitive impairment were also excluded.

The study involved administering a structured, interviewer-guided questionnaire to both the intervention and control groups at two key time points: before the educational program began (baseline) and six months after its completion (post-intervention) to capture the participants' initial status and assess any changes resulting from the intervention over a longer period.

Trained interviewers read the questions aloud and recorded participants' responses, ensuring inclusivity for those with limited literacy skills. This method minimized barriers to data collection and reduced the risk of incomplete or inaccurate responses due to literacy challenges, enabling full participation.

### Participant group allocation

Participants were assigned to either the intervention or control group based on the pre-assigned hospital grouping, ensuring that each hospital was designated to one group to avoid interference in the study outcomes.

Intervention Group (*n* = 102): This group received small-group educational sessions covering topics such as the nature of asthma, identifying triggers, environmental control, medication adjustments, and managing exacerbations. The sessions were delivered uniformly across hospitals to ensure consistency in duration and frequency. In addition to the education, participants also received standard care provided at their respective hospitals.

Control Group (*n* = 102): This group received only the routine standard asthma care offered at the hospitals, without any additional educational intervention.

### Outcome measures

#### Asthma control level

Asthma control level was assessed using the Asthma Control Test, a widely used and validated tool in global asthma research. This tool consists of five items that assess the presence or absence of nocturnal symptoms, daytime symptoms (coughing, chest tightness, and wheezing), use of rescue medications, symptom interference with daily activities, and absenteeism from work or school. Responses to these five items are summed to yield a score ranging from 5 (poorly controlled) to 25 (completely controlled). Scores are classified as follows: 20–25 = well-controlled asthma, 16–19 = partially controlled asthma, and 5–15 = uncontrolled asthma. Higher scores indicate better control, and a score of 19 or less is considered a cut-off for poor control based on the previous literatures [32, 35]. These three levels were then converted to percentiles for linear regression analysis to examine variations across groups. The internal consistency, as measured by Cronbach’s Alpha, was found to be 0.72, suggesting good reliability, while the test–retest reliability, assessed using the Intraclass Correlation Coefficient (ICC), was 0.63, indicating moderate stability over time [1–3]. Although the Asthma Control Test has not been specifically validated in Ethiopia, it has been widely used in asthma populations globally, providing a reliable measure of asthma control.

#### Self-management knowledge level

Self-management knowledge was assessed using the Asthma Self-Management Questionnaire (ASMQ), which is designed to evaluate patients' knowledge about asthma and its management [36]. While the standard ASMQ includes 16 questions assessing protective awareness, inhaler use, medication use (rescue and control), and peak flow meter use (Mancuso et al., 2009), two questions related to the peak flow meter were excluded from this study due to their limited applicability in the Ethiopian context. The tool's scores were calculated by assigning one point for each preferred response, and the points were then summed to yield a raw score ranging from 0 to 14. The items were phrased as questions with four response options, and the scores were recoded to produce a final result out of 14. The mean scores across all items were calculated, with higher scores indicating better knowledge. The raw score was then converted to a scale of 0–100, with higher scores reflecting a higher level of knowledge. Knowledge levels were categorized as follows: i) Good knowledge (ASMQ > 75 transformed), ii) Adequate knowledge (transformed ASMQ = 50–75), and iii) Poor knowledge (transformed ASMQ < 50), based on previous literature [36, 37]. Reliability testing revealed an internal consistency (Cronbach’s Alpha) of 0.61, suggesting fair reliability, and the test–retest reliability, assessed using ICC, was 0.58, indicating fair stability over time [4]. Although the ASMQ has not been validated in Ethiopia, it has been widely used in other studies globally [14, 36].

### Educational intervention methods

The asthma self-management education (ASME) program consisted of three interactive sessions for participants in the intervention group, held at baseline (the first contact), 3 months, and 6 months. Each session lasted one to two hours and covered essential topics such as the nature of asthma, appropriate use of inhaled medications, environmental control strategies, and self-monitoring skills. The aim of the educational sessions was to enhance participants' understanding of asthma, improve self-management practices, and empower them to manage their condition more effectively.

The educational content was adapted from existing literature, and facilitators were provided with manuals from the same sourced from [13, 38–40]. They received 3 days of training on asthma basics and the specific material to be taught. These facilitators were healthcare professionals with BSc degree in health science, not asthma experts. Leaflets that summarized key points from the sessions were prepared in the local language, Tigrigna, to use it as a reminder at home. The teaching methods included lectures, group discussions, and demonstrations. Each group included small group consisting 13 to 16 patients.

The educational program was delivered consistently across the selected hospitals. In addition to the educational intervention, participants continued to receive the standard care provided at their respective hospitals. Data were collected at baseline and again 6 months after the completion of educational intervention from both intervention and control groups. Of the 204 participants initially recruited, 45 were lost to follow-up at various stages: 8 at 3 months, 13 at 6 months, and 24 during the post-intervention period, leaving a final sample of 159 participants.

### Demographics

Both intervention and control groups provided socio-demographic and asthma-related information, including age, gender, education, residence, asthma duration, smoking history, and triggers.

#### Statistical analysis

Baseline (intervention and control groups) and post-test data (intervention and control groups) were combined into a single dataset to examine changes in asthma control and self-management knowledge over time. Differences in socio-demographic and asthma-related variables (e.g., age, gender, marital status, education, occupation, income, asthma duration, smoking history, and asthma triggers) between the intervention and control groups were assessed using chi-square tests, which showed no significant baseline or post-test differences, ensuring that these variables did not confound the intervention's evaluation.

For the primary analysis, linear regression was used to compare differences in asthma control and self-management knowledge between groups. A time variable (baseline = 0, post-test = 1) and a program variable (intervention = 1, control = 0) were created, along with a time-program interaction term to assess the intervention's effects. The Difference-in-Differences (DiD) approach was employed, assuming parallel trends between the groups in the absence of the intervention. This approach accounted for pre-existing trends and provided an estimate of the intervention's impact through the unstandardized beta coefficient (β) of the interaction term, with statistical significance set at *p* value < 0.05.

Covariates, including sex, age, educational status, income, asthma duration, and smoking status, were incorporated to adjust for potential confounders, and clustering of patients within hospitals was accounted for in the analysis. A heterogeneity analysis further examined variations in the intervention's effects across subgroups defined by sex, age, income, and disease duration. All analyses and computations were performed using SPSS version 25.

## Result

The study included 183 participants in the intervention group and 180 in the control group, with a loss to follow-up of 20.6% and 23.5%, respectively. Males comprised 65% of the participants, 40% were aged 35–64, and nearly half of the intervention group and a quarter of the control group were 65 years or older.

More than half of the participants in both groups were married, with 16% and 19% of the intervention and control groups, respectively, being illiterate. About 20% in each group were unemployed, 70% were urban residents, and 17.6% of the control group and 29.4% of the intervention group reported high income. (See Table 1 for demographic information).

**Table 1:** Socio-economic characteristics of study participants in Tigray Region, 2020 (*n* = 363)

Variables	Baseline		Post Intervention		Chi square	*P* value
	Intervention (*n* = 102)	Comparison(*n* = 102)	Intervention (*n* = 81)	Comparison (*n* = 78)		
	n (%)	n (%)	n (%)	n (%)		
**Sex**					**0.199**	**0.656**
Male	70 (68.6)	64 (62.7)	51 (63.0)	51 (65.4)		
Female	32 (31.4	38 (37.3)	30 (37.0)	27 (34.6)		
**Age group (years)**					**5.252**	**0.072**
18–34	15(14.7)	24 (23.5)	11 (13.6)	15 (19.2)		
35–64	38 (37.3)	44(43.1)	31 (38.3)	29 (37.2)		
65 and above	49 (48)	34(33.3)	39 (48.1)	34 (43.6)		
**Marital status**					**2.94**	**0.40**
Married	63(61.8)	53 (52.0)	50 (61.7)	46 (59)		
Single	20(19.6)	35 (34.3)	15 (18.5)	19 (24.4)		
Divorced	11(10.8)	7 (6.9)	10 (12.3)	7 (9)		
Widowed	8( 7.8)	7 (6.9)	6 (7.4)	6(7.7)		
**Education**					**3.35**	**0.34**
Illiterate	16( 15.7)	19 (18.6)	13 (16)	14 (17.9)		
Primary school	28( 27.5)	27 (26.5)	22 (27.2)	21 (26.9)		
secondary school	37( 36.3)	40 (39.2)	32 (39.5)	31 (39.7)		
Diploma and above	21( 20.6)	16 (15.7)	14 (17.3)	12 (15.4)		
**Occupation**					**6.74**	**0.08**
Unemployed	23 (22.5)	30 (29.4)	18 (22.2)	23 (29.5)		
Farmer	22 (21.6)	30 (29.4)	21 (25.9)	26 (33.3)		
Merchant	33 ( 32.4)	17 (16.7)	25 (30.9)	17 (21.8)		
Civil servant	24 ( 23.5)	25 (24.5)	17 (21.0)	12 (15.4)		
**Residence**					**2.66**	**0.10**
Urban	72 (70.6)	61 (59.8)	58 (71.6)	42 (53.8)		
Rural	30 (29.4)	41 (40.2)	23 (28.4)	36 (46.2)		
**Income**					**5.59**	**0.061**
less than 1000	25 (24.5)	20 (19.6)	18 (22.2)	17 (21.8)		
1000–4000	47(46.1)	64 (62.7)	38(46.9)	42 (53.8)		
greater than 4000	30(29.4)	18(17.6)	25 (30.9)	19 (24.4)		

### Asthma-related characteristics

Most participants had asthma for over 10 years. Ten percent of the intervention group and 7.8% of the control group had asthma education, mostly over ten years ago. Smoking history was reported by 10% of the intervention group and 14% of the control group, with cold weather and dust as common triggers (See Table 2).

**Table 2:** Asthma related characteristics of study participants in Tigray Region, 2020 (*n* = 363)

Variables	Baseline		Post intervention		Chi square	*P* value
	Intervention (*n* = 102)	Comparison (*n* = 102)	Intervention (*n* = 81)	Comparison (*n* = 78)		
	n (%)	n (%)	n (%)	n (%)		
**Duration of disease (years)**					**0.595**	**0.44**
1–10 years	37 (36.3)	43 (42.2)	32 (39.5)	32 (41)		
above 10 years	65 (63.7)	59 (57.8)	49 (60.5)	46 (59)		
**Received asthma education**					**0.449**	**0.501**
Yes	11 (10.8)	8 (7.8)	8 (9.9)	7 (9)		
No	91 (89.2)	94 (92.2)	73 (90.1)	71 (91)		
**Time of asthma education**					**0.941**	**0.332**
below 10 years	5(4.9)	2(2)	4 (4.9)	3 (3.8)		
above 10 years	6(5.9)	6 (5.9)	6 (7.4)	1 (1.3)		
**History of smoking**					**0.297**	**0.586**
No	92 (90.2)	88 (86.3)	71 (87.7)	69 (88.5)		
Yes	10 (9.8)	14 (13.7)	10 (12.3)	9 (11.5)		
**Asthma triggers as reported by the study participants**					**5.574**	**0.350**
Cold weather	26( 25.5)	28 (27.5)	20 (24.7)	21 (26.9)		
Dust	23(25.5)	29 (28.4)	19 (23.5)	26 (33.3)		
Strong smell ( spray, perfume)	14( 13.7)	13 (12.7)	12 (14.8)	9 (11.5)		
Smoke	20(19.6 )	20 (19.6)	17 (21)	13 (16.7)		

### The effect of the educational intervention

#### Asthma control level

Asthma control significantly improved in the intervention group, with well-controlled asthma increasing from 27.5% at baseline to 46.9% post-intervention (19.4% increase). In contrast, the control group showed a minimal change, from 22.5% to 23.1% (0.6% increase). The intervention group showed 18.8% greater improvement in asthma control (Difference-in-Difference, DiD). (See Table 3).

**Table 3:** Change between baseline and end line of asthma control level between the comparison group in Tigray Region, 2020 (*n* = 363)

Asthma control levels	Time	Intervention (%)	Control n (%)	Difference%
Well controlled (> 19)	Baseline	28(27.5)	23(22.5)	5
	End-line	38(46.9)	18(23.10)	23.8
	Difference %	19.4	0.6	**18.8 (DiD)**

In the intervention group, the proportion of participants with poorly controlled asthma decreased significantly, from 56.9% to 38.3%. The proportion with partially controlled asthma showed only a slight decline, from 15.7% to 14.8%. In contrast, the control group experienced a slight increase in the proportion of participants with poorly controlled asthma, from 22.5% to 24.4%, while the proportion with partially controlled asthma decreased from 54.9% to 52.6%.

After adjusting for potential confounders, the Difference-in-Difference (DiD) analysis revealed a significant improvement in asthma control in the intervention group compared to the control group. The linear regression analysis indicated that asthma control levels were 6.3 times higher in the intervention group than in the control group (*P* = 0.03), after adjusting for sex, age, education, income, and disease duration. None of the covariates, including sex (*P* = 0.448), age (*P* = 0.568), education (*P* = 0.466), income (*P* = 0.999), or disease duration (*P* = 0.467), showed statistically significant associations with asthma self-management knowledge (See Table 4).

**Table 4:** Estimating effect of the intervention on asthma control level between study groups after adjusting for other variables in Tigray Region, 2020 (*n* = 363)

Variables	Unstandardized coefficients	T	95% CI for B		*P* value
			Lower	Upper	
Time and Program Interaction	6.305	2.160	.611	12.000	0.030*
Sex	1.557	1.007	−1.485	4.598	.448
Age	-.974	-.951	−2.988	1.039	.568
Education	.353	.463	−1.145	1.850	.466
Income	-.002	-.002	−2.061	2.058	.999
Disease Duration	−1.073	-.728	−3.970	1.825	.467

^*^ Statistically significant

#### Asthma self-management knowledge level

Asthma self-management knowledge significantly increased in the intervention group, but not in the control group. In the intervention group, the proportion of participants with good knowledge increased from 2.9% at baseline to 27.2% post-intervention, representing a 24.3% increase. In contrast, the control group showed a minimal increase, with 2% having good knowledge at baseline and 2.7% post-intervention, resulting in a 0.7% increase. Overall, the intervention group demonstrated a 23.5% greater improvement (DiD) compared to the control group ( See Table 5).

**Table 5:** Change in asthma self-management knowledge levels between baseline and post-intervention in the comparison group in Tigray Region, 2020 (*n* = 363)

Knowledge levels	Time	Baseline n (%)	Posttest n(%)	Difference %
Good (> 75%)	Baseline	3(2.9)	2(2)	0.9
	Posttest	22 (27.2)	3 (2.7)	24.5
	Difference %	24.3	0.7	**23.6 (DiD)**

Breaking it down by knowledge levels, the proportion of participants in the intervention group with poor knowledge decreased from 86 to 42%, while those with adequate knowledge increased from 10.8% to 30.9%. The control group experienced minimal changes, with poor knowledge decreasing slightly from 81% to 80.8%, and the proportion with adequate knowledge remaining stable at 16.7%.

After controlling for potential confounders, the DiD analysis showed a significant difference in asthma self-management knowledge level between the intervention and control groups. The linear regression unstandardized coefficient result showed that the likelihood of having good asthma self-management knowledge among participants in the intervention group was 13.3 times higher than in the control group (13.338, 95% CI: 6.717–19.959, *P* < 0.001), after adjusting for sex, age, education, income, and duration of disease. None of the covariates, including sex (*P* = 0.052), age (*P* = 0.163), education (*P* = 0.933), income (*P* = 0.762), or disease duration (*P* = 0.176), showed statistically significant associations with asthma self-management knowledge, suggesting the observed improvement was primarily attributable to the intervention (Table 6).

**Table 6:** Estimating the effect of the intervention on asthma self-management knowledge level between study groups after adjusting other variables in Tigray Region, 2020(*n* = 363)

Variables	Unstandardized coefficients	T	95% CI for B		*P* value
			Lower	Upper	
Time and Program Interaction	13.338	3.996	6.717	19.959	.000**
Sex	−3.500	−1.946	−7.036	.037	.052
Age	1.664	1.398	-.677	4.006	.163
Education	-.074	-.084	−1.815	1.667	.933
Income	.370	.304	−2.025	2.764	.762
Disease Duration	−2.325	−1.357	−5.694	1.044	.176

^**^ Highly statistically significant

#### Heterogeneity analysis

Heterogeneity analysis revealed no significant differences in the effects of the educational intervention on asthma control and self-management knowledge across subgroups defined by sex, age, income, and disease duration. For asthma control, the interaction term was not significant (F = 2.092, *P* = 0.070), with the model explaining 55.7% of the variance (adjusted R2 = 26.9%). Similarly, for self-management knowledge, the interaction term was also not significant (F = 1.516, *P* = 0.198), with 47.7% of the variance explained (adjusted R2 = 13.7%) (See Table 7).

**Table 7:** Heterogeneity in effect of the interventions on gender, age, income and disease duration after adjusting for other variables in Tigray Region, 2020(*n* = 363)

Variable	Asthma control		Knowledge level		Asthma control		Knowledge level	
	F value	*P* value	F value	*P* value	R^2^	Adjusted R^2^	R^2^	Adjusted R^2^
Sex, age, income &disease duration	2.092	0.070	1.516	0.198	0.557	0.269	0.477	0.137

## Discussion

The study targeted asthma patients who were not hospitalized but visited the hospital regularly for routine check-ups and medication refills. Different hospitals use various names for these follow-up services, such as "asthma clinics," "chronic care" services, or simply the outpatient department. Despite the different terminology, the focus of these settings remains the same: providing ongoing care and support for patients managing long-term conditions like asthma. These patients were selected to evaluate the outcomes of providing small-group asthma education within outpatient settings, where most ongoing asthma management occurs.

By increasing self-management knowledge, patients are better able to control their asthma symptoms, improving their level of asthma control. In addition to taking their medications as prescribed, patients can effectively reduce the costs and harm associated with uncontrolled and poorly managed asthma. Therefore, the aim of the current study was to evaluate the effect of self-management education on the level of asthma control and self-management knowledge among adult patients with asthma.

The data in the current study demonstrated a significant gap in asthma education within healthcare settings, with only 10% of the intervention group and 7.8% of the comparison group reporting receiving asthma-related information from pharmacies or outpatient departments (OPDs), most of which occurred over a decade ago. This suggests that asthma education is not prioritized in routine healthcare practices. Research indicates that when healthcare providers integrate asthma education into their care routines, it leads to better asthma management and improved patient outcomes. For instance, a study found that patients whose healthcare providers participated in asthma care education programs experienced a greater reduction in asthma symptoms and fewer emergency department visits compared to those who did not receive such education [41].

Integrating asthma education into standard care is likely to improve health outcomes for asthma patients. Education equips patients with critical skills, such as problem-solving, self-efficacy, and better control over their condition. These skills enable patients to adhere to treatment plans, manage triggers, and respond to symptoms effectively. Additionally, structured asthma education has been shown to improve asthma control and self-management, with one study finding that participants who received education scored higher in asthma control and knowledge [42]. Furthermore, asthma education fosters learned effectiveness, empowering patients to actively manage their health, which is associated with better disease control and reduced morbidity. Therefore, incorporating asthma education into healthcare practices could significantly improve patient outcomes.

By integrating structured self-management education programs into OPDs settings, healthcare providers could reduce the burden of asthma, improving patients' quality of life and reducing healthcare costs associated with emergency visits, hospitalizations, and complications from poorly managed asthma. This study assessed the impact of an asthma education intervention on patients attending follow-up visits in hospital OPDs and chest clinics. The intervention included three introductory sessions on asthma self-management and control strategies. Participants demonstrated statistically significant improvements in both asthma control and self-management knowledge, showing the program's effectiveness.

At baseline, most respondents in both the intervention and comparison groups had poor asthma control levels. This similarity in baseline asthma control is crucial for ensuring the validity of the study findings, as it allows us to isolate the effect of the educational intervention on outcomes. Our results are consistent with those of the study Impact of Therapeutic Education on Asthma Control, Medication Adherence, Knowledge and Quality of Life in Moroccan Adult Asthma Patients, which also reported no significant difference in asthma control test scores between intervention and comparison groups at baseline [43]. This baseline comparability reinforces the relevance of the intervention, as it demonstrates that both groups started from an equal footing, allowing us to attribute any observed differences in outcomes to the intervention itself rather than pre-existing disparities.

After post-intervention, the asthma control level in the intervention group significantly increased. The intervention group had higher well-controlled asthma scores than the comparison group. The significant improvement in asthma control levels in the intervention group suggests that the asthma education intervention played a key role in enhancing patient outcomes. Previous studies have shown that asthma education can empower patients to manage their condition more effectively by increasing self-efficacy, knowledge, and adherence to treatment plans. In this study, the intervention group’s exposure to repeated small-group education may have contributed to a better understanding of asthma management, leading to improved asthma control. The six-fold higher likelihood of achieving well-controlled asthma in the intervention group aligns with findings from similar interventions that emphasize self-management education. Furthermore, the reduction in poorly controlled asthma cases from 56.9% to 38.3% underscores the effectiveness of education in improving long-term disease management. This reduction is consistent with literature that supports asthma education as an effective strategy to reduce symptoms and prevent complications associated with uncontrolled asthma. These findings are consistent with Mishra R et al. (2017) and Boulet L-P et al. (2015), who showed that asthma control levels significantly improved after an asthma education program [44, 45]. Similar evaluations have also been observed in studies by Poureslami I et al. (2012), Mammen JR et al. (2018), Toppila-Salmi S et al. (2021), Kavut AB and Kalpaklıoğlu AF (2010), and Ghaleb Dailah & Hamad (2021) which showed a positive relation between asthma control level and self-management education [12, 18, 42, 46, 47].

On the other hand, a study by Felix SN et al. (2021) found no significant differences in control levels [48]. Another study by Baptist AP et al. (2010) showed no differences in short-term and long-term asthma outcomes between older adults in the control and intervention groups [49]. However, the current study focuses only on the evaluation of the final outcome without specifying the terms of the outcome. This discrepancy may result from differences in the study population, duration, and interval of the education provided.

Regarding knowledge level, most respondents had low asthma self-management knowledge levels in both the intervention and comparison groups at baseline there was no significant difference in knowledge between the two study groups, which reflected their homogeneity. This is in line with several studies who reported that most of the study subjects had unsatisfactory asthma knowledge before implementing the educational program [37, 50].

After post-intervention, in the intervention group asthma self-management knowledge level was significantly increased. The intervention group had higher self-management knowledge scores than the comparison group in all four domains of the knowledge questionnaire. The significant improvement in self-management knowledge in the intervention group underscores the effectiveness of the asthma education program. Respondents in the intervention group had 13 times higher levels of good knowledge than those in the comparison group, with a substantial decrease in the proportion of patients with poor self-management knowledge (from 86 to 42%). This suggests that structured education programs can significantly enhance patients' understanding of their condition, which is crucial for managing asthma effectively. Improved knowledge in key areas, such as symptom recognition, medication adherence, and trigger management, likely empowers patients to take a more active role in managing their asthma. This, in turn, may lead to better control of asthma symptoms and a reduction in the risk of acute exacerbations. The reduction in patients with poor knowledge levels further suggests that educational interventions may be pivotal in bridging gaps in asthma care. Our study reproduced previously reported findings from interventional quasi-experimental and randomized controlled trials, which evaluated the positive impact of educational programs on asthma self-management knowledge. Similar results were observed in studies by Ali A et al. (2019), Manchana V and Mahal RK (2014), and Elbanna RM et al. (2017), all of which revealed significant improvements in patient knowledge following the implementation of self-management programs [28, 51, 52]. Furthermore, our results align with studies by Poureslami I et al. (2012), Huang TT and Li YT (2009), El-Fadl NMA and Sheta HAE-S(2023), Behera Kaur et al. (2006), and Ghaleb Dailah H (2021) which reported highly statistically significant differences between study and control groups pre-post and at follow-up after implementing self-learning modules [42, 43, 47, 53, 54].

On the other hand, sex, age, education, income, duration of disease, previous asthma education, identifying asthma triggers, and smoking history were not significantly associated with both outcomes in the linear regression analysis (*p* > 0.05 for all variables). This finding contrasts with the results of Mammen JR et al. (2018)), which reported significant differences in mean change scores by sex, with greater improvement in knowledge found in females than males [12]. Another study also mentioned that women were much more likely to be educated about asthma control and management and more likely to carry a rescue inhaler than men as far as literacy level is concerned [55]. The observed discrepancy could be due to differences in the study population, study design, and the evaluation period, which were not considered in the current study.

This study highlights the importance of integrating structured asthma education into routine outpatient care. By enhancing self-management knowledge, patients can better control their asthma, reducing the need for emergency visits and hospitalizations. These interventions can significantly lower healthcare costs and improve patient outcomes. Bridging the gap in asthma education is crucial for optimizing asthma management and improving patients' overall QOL.

This study highlights the critical role of asthma education in improving asthma control and self-management knowledge. The findings demonstrate that participants in the intervention group achieved significantly higher asthma control levels and self-management knowledge compared to the control group. These improvements align with existing literature that emphasizes the positive impact of structured educational programs on patient outcomes. However, the descriptive analysis revealed a significant gap: many healthcare facilities still do not routinely integrate asthma education into standard care.

The results of this study showed the importance of incorporating structured asthma education into routine outpatient care. Such programs not only enhance self-management knowledge but also enable patients to better control their asthma, which can reduce the need for emergency visits, hospitalizations, and related complications. By addressing this educational gap, healthcare providers can optimize asthma management and improve patients' overall quality of life.

### Limitations and strengths

This quasi-experimental study has some limitations. The use of convenience and purposive sampling may limit generalizability, and the absence of random assignment could affect internal validity. However, baseline similarity between the intervention and control groups likely minimized major selection bias.

Despite the above limitations, the study has notable strengths. The inclusion of a control group provides a strong basis for comparison, reinforcing the validity of the findings. The adequate sample size ensured sufficient statistical power to detect significant changes in outcomes, even with some attrition. Conducting the intervention in real-world outpatient settings enhances the external validity and practical relevance of the results. Moreover, pre- and post-intervention assessments offered evidence of the intervention’s effectiveness, supporting its value in improving asthma control and self-management knowledge. Future research which prioritize random sampling and randomized designs can enhance both internal and external validity.

## Conclusion and recommendation

Asthma education plays a critical role in improving asthma outcomes by enhancing asthma control levels and increasing self-management knowledge. The findings of this study demonstrate that, post-intervention, participants in the intervention group showed significantly higher asthma control and self-management knowledge compared to the comparison group. These improvements were statistically significant, demonstrating the effectiveness of structured asthma education. Furthermore, the demographic analysis revealed that many healthcare facilities do not routinely offer asthma education as part of standard care, indicating a significant gap in patient management.

To address this gap, expanding asthma education programs as a core component of follow-up visits is essential. These programs can help reduce medical costs, minimize emergency department visits, and lower asthma-related mortality. Therefore, healthcare providers should prioritize asthma education in their care protocols to improve patient outcomes and enhance the overall quality of asthma management.

## Supplementary Information

Supplementary Material 1.

## Data Availability

The datasets used or analyzed during the current study are available from the corresponding author upon reasonable request.

## Abbreviations

ACT: Asthma Control Test
ASMQ: Asthma self-management questionnaire
QOL: Quality of life
ASME: Asthma self-management education
ED: Emergency department
DID: Difference in difference
CI: Confidence interval
SPSS: Statistical packages for social sciences
