# Supplementary material for: Effect of asthma education intervention on self-management knowledge and control level in Tigray, Northern Ethiopia: a quasi experimental study
Source: BMC Pulm Med. 2025 Mar 15;25:120. doi: 10.1186/s12890-025-03574-4 (PMC11910835; doi:10.1186/s12890-025-03574-4)
Supplement: Supplementary file 1 — Supplementary Material 1. [file 12890_2025_3574_MOESM1_ESM.docx]

| Hospital ID |  |  |  |  |  |
| --- | --- | --- | --- | --- | --- |
| Interviewer ID |  |  |  |  |  |
| Participant ID |  |  |  |  |  |
| Date |  |  |  |  |  |
| My name is ______________________ I would like to fill this questionnaire today within 30 minutes on effect of asthma self-management education on asthma outcomes regarding your medical condition while you are doing your follow up activities. Confidentiality regarding this interview will be strictly maintained. At any time during our interview, feel-free to ask if you have any questions and you can stop the interview at any time if you do not want or you can escape any question which is (are) not-comfortable for you.  Are you volunteer to participate in this study?   1. Yes 2. No ( write the reason and continue to the next participant ) | | | | | |

| **Part 1: Socio Demographic Related Questions** | | | |
| --- | --- | --- | --- |
| **No.** | **Questions** | **Alternative choice for respondents** | |
| 101 | Sex | 1. Male 2. Female | |
| 102 | Age | ________ years | |
| 103 | Religion | \| 1 \| Orthodox \| \| --- \| --- \| \| 2 \| Muslim \| \| 3 \| Others \| | |
| 104 | Ethnicity | \| 1. Tigrian \| \| --- \| \| 1. Other specify \| | |
| 105 | Marital status | \| 1. Married \| \| --- \| \| 1. Single \| \| 1. Divorced \| \| 1. Widow \| | |
| 106 | Educational status | 1. Illiterate 2. Primary school 3. Secondary school 4. Diploma and above | |
| 107 | Employment status | \| 1. Unemployed \| \| --- \| \| 1. Farmer 2. Merchant 3. Civil servant \| | |
| 108 | Residence | \| 1. Urban \| \| --- \| \| 1. Rural \| | |
| 109 | Income | 1. Urban 2. Rural | |
| 110 | Duration of disease in years | 1. Below 10 years 2. Above 10 years | |
| 111 | Did you get any asthma self-management related education before? | 1. No 2. Yes | |
| 112 | If your answer is yes to Q111, when was the time of asthma education provided | 1. Below 10 years 2. Above 10 years | |
| 113 | History of smoking | 1. No 2. Yes | |
| 114 | What are your asthma triggers | 1. Cold weather 2. Dust 3. Strong smell 4. Smoke 5. Others (chemicals, pollens) 6. I do not know | |
| **Part II: Asthma symptom control Related questions (ACT)** | | | |
| 201 | In the past 4 weeks, how much of the time did your asthma keep you from getting as much done at work, school or at home | \| 1. All of the time 2. Most of the time 3. Some of the time 4. Little of the time 5. None of the time \| \| --- \| | |
| 202 | During the past 4 weeks, how often have you had shortness of breath? | 1. more than a day 2. Once a day 3. 3-6 times per week 4. Once or twice a week 5. Not at all | |
| 203 | During the past 4 weeks, how often did your asthma symptoms (wheezing, coughing, chest tightness or pain wake up at night or earlier than usual in the morning? | 1. more than a day 2. Once a day 3. 3-6 times per week 4. Once or twice a week 5. Not at all | |
| 204 | During the past 4 weeks, how often have you used your rescue inhaler (such as albuterol)? | 1. 3 or more times per day 2. 1 or 2 times per day 3. 2 or 3 times per week 4. One a week or less 5. Not at all | |
| 205 | How would you rate your asthma control during the past 4 weeks? | 1. Not controlled at all 2. Poorly controlled 3. Somewhat controlled 4. Well controlled 5. Completely controlled | |
| **Part III: Asthma self-management knowledge related questions (ASMQ)** | | | |
| 301 | Taking the prescribed two puffs of  your inhaler two times a day: | 1. Same as taking one puff four times a day 2. Same as taking four puffs once a day 3. Be rearranged in anyway as long as you take four times a day 4. Is not the same as any other regimen 5. I do not know |  |
| 302 | If you are not having asthma symptoms… | 1. Your lungs are not sensitive to irritants 2. It is ok so skip some doses of medicine 3. You should still avoid triggers 4. You are probably cured of asthma 5. I do not know |  |
| 303 | Maintenance medicines… | 1. Maintenance medicines… 2. Do not need to be taken every day 3. Make you breath better right after you take them 4. Can only be taken in pill form 5. I do not know |  |
| 304 | Rescue medicines | 1. Should not be taken more than three or four times a day 2. Help prevent future flare ups 3. Have no side effects 4. Do not cause you to become tolerant to medicines 5. I do not know |  |
| 305 | When using your inhaler, you should | 1. When using your inhaler, you should 2. Inhale quickly 3. Inhale slowly 4. press your inhaler several times while you are inhaling 5. I do not know |  |
| 306 | After you have used your inhaler, you should | 1. Hold your breath for several seconds 2. second puff as soon as possible after the first puff 3. Keep taking puffs until you feel better 4. Wash the inhaler in a tub of water 5. I do not know |  |
| 307 | If you are having symptoms and do not know why the first thing you should do is: | 1. Take some doses of steroid medicine 2. Visit health facility 3. Change your immediate environment 4. Take traditional medicine 5. I do not know |  |
| 308 | What does it mean that taking more rescue inhaler than prescribed is? | 1. Is really not harmful 2. Good way to manage symptoms caused 3. May mean you can take less maintenance 4. May mean you can take more maintenance 5. I do not know |  |
| 309 | What is the role of exercise on asthmatic patients? | 1. Something that should not be done regularly 2. Can help improve breathing capacity 3. Is only good if done for at least 30 minutes 4. Can trigger symptoms because the lungs are not taking in enough oxygen 5. I do not know |  |
| 310 | Asthma can be cured by | 1. Taking daily medicine 2. Avoiding triggers such as dust and cigarette smoking 3. There is no known cure of asthma 4. I do not know |  |
| 311 | What are the warning signs of asthma worsening (exacerbation or flare-ups) | \| 1. Usually occur suddenly without warning 2. Occurs when several minor triggers come together 3. Cannot be triggered by strong emotions 4. Always cause wheezing 5. I do not know \| \| --- \| |  |
| 312 | If you are prescribed a seven-day course of steroid pills | 1. You do not have to avoid triggers while taking the pills 2. Your symptoms can not get worse while taking the pills 3. You should finish the prescription even if you feel better after taking several doses 4. I do not know |  |
| 313 | Which of the following can help control asthma? | \| 1. Reducing stress level 2. Drinking plenty of water to stay hydrated 3. Avoiding foods like dried fruits & wine 4. All of the above 5. I do not know \| \| --- \| |  |
| 314 | What is the main measure you take to prevent asthma flare ups | 1. Take medicines before meals as prescribed 2. Go to emergency room at the first sign of symptoms 3. Take steroids in pills form 4. I do not know |  |
